# Supplementary material for: Rapid turnover of life-cycle-related genes in the brown algae
Source: Genome Biol. 2019 Feb 14;20:35. doi: 10.1186/s13059-019-1630-6 (PMC6374913; doi:10.1186/s13059-019-1630-6)
Supplement: Supplementary file 3 — Protein alignments, corresponding Gblocks html file and CDS sequences for the lineage conserved gametophyte-biased genes. (ZIP 2109 kb) [file 13059_2019_1630_MOESM3_ESM.zip › Con_GA_alignments/Ec-08_001650.aa_ali.fasta-gb.htm]

Ec-08\_001650.aa.fasta.trans.aa\_ali.fasta


## Gblocks 0.91b Results

Processed file: **Ec-08\_001650.aa.fasta.trans.aa\_ali.fasta**  
Number of sequences: **2**  
Alignment assumed to be: **Protein**  
New number of positions: **205** (selected positions are underlined in blue)

```
                         10        20        30        40        50        60
                 =========+=========+=========+=========+=========+=========+
Ec-08_001650     MSDKQGQGHLGVGKPSVADEKGGTSGGGVSGDDGDARASRDREDGRPACLPLPSPPRLPS
MSTRG.30795.1    MSKESGPGHLGVDDPPSTQRQSESVEG--SGGDGGAGP-RSGEAGRPVCLPLPSTPRLPS
                 ###############                        #####################


                         70        80        90       100       110       120
                 =========+=========+=========+=========+=========+=========+
Ec-08_001650     KRTLTDSVMDSTVARFEASCKLTEQGT--AAAPSAARTALATVSSAANTVRTPSWDKNAT
MSTRG.30795.1    KRTIKSSCVDDTAAQFEAKCTVGSGRTTNKAVPSRGMADMVAAALEATAVRLPSWDK-ST
                 ###########################                   ###########  #


                        130       140       150       160       170       180
                 =========+=========+=========+=========+=========+=========+
Ec-08_001650     PAHLEARERTAQAWIEAVSGRACVEYQTKQFARNLMSGEILCALVNAIRPGTIPHVHESQ
MSTRG.30795.1    PADLEARERTAQVWIEAVSGKPCVAYETRQFARNLKSGEILCALVNAIRPGTIPVVHEGQ
                 ############################################################


                        190       200       210       220       230       240
                 =========+=========+=========+=========+=========+=========+
Ec-08_001650     LGFEQMENINSFLSACCKLGVQAHALVDTADVFEMRDTAKVVECVHALGSAVQVSCPEFG
MSTRG.30795.1    LGFNQMENVDNFLSACCKLGVPAHALVDTADVFEMRDTAKLVECVHALGSVVQVTCPGFR
                 ############################################################


                        250       260       270       280       290       300
                 =========+=========+=========+=========+=========+=========+
Ec-08_001650     GPHLGPAADPTLVAAVPPADEDEGDDGGDGDGKDNAVADDDDDEDGAFGDFWRASVRTGG
MSTRG.30795.1    GPHLGLAADPX-------------------------------------------------
                 ##########                                                  


                        310       320       330       340       350       360
                 =========+=========+=========+=========+=========+=========+
Ec-08_001650     NAPREGGGGRDGDVAGDNTLVLNMQDVERPRSTRRGFTTTVGSITDLDPPATLSARARQM
MSTRG.30795.1    ------------------------------------------------------------
                                                                             


                        370       380       390       400       410       420
                 =========+=========+=========+=========+=========+=========+
Ec-08_001650     CPTEMVNSPRALVFNADPVGHPTRSPTLRAAWESNATNGGRAAAGRSAWGTDAYPAKRAA
MSTRG.30795.1    ------------------------------------------------------------
                                                                             


                        430       440       450       460       470       480
                 =========+=========+=========+=========+=========+=========+
Ec-08_001650     KATSNLSATSSRGMTLSPAGIWGTGAGTAPREGIGKSASPAAASANSATAAAVAASHTGS
MSTRG.30795.1    ------------------------------------------------------------
                                                                             


                        490       500
                 =========+=========+=
Ec-08_001650     EDPEGTEEGAEETGGGGGGRR
MSTRG.30795.1    ---------------------
```

```
Parameters used
Minimum Number Of Sequences For A Conserved Position: 2
Minimum Number Of Sequences For A Flanking Position: 2
Maximum Number Of Contiguous Nonconserved Positions: 8
Minimum Length Of A Block: 10
Allowed Gap Positions: None
Use Similarity Matrices: Yes
```

```
Flank positions of the 4 selected block(s)
Flanks: [1  15]  [40  87]  [107  117]  [120  250]  

New number of positions in Ec-08_001650.aa.fasta.trans.aa_ali.fasta-gb:  205  (40% of the original 501 positions)
```
